# Reliability of Ultrasound Measurements in the Assessment of Femoral Trochlear Morphology in Healthy Adolescents

**DOI:** 10.3390/diagnostics16091381

**Published:** 2026-05-01

**Authors:** Stefan A. Djordjevic, Milica Vitomirac, Bojan Bukva, Gordana Susic, Smiljka Kovacevic, Dragana Lazarevic, Ana Cirovic, Hristina Petrovic, Maja Bijelic, Tijana Dimkic-Tomic, Andjela Dimkic-Milenkovic, Vladimir Milenkovic, Goran Djuricic

**Affiliations:** 1 Division of Pediatric Rheumatology, University Children’s Hospital, 11000 Belgrade, Serbia; milicavitomirac@gmail.com (M.V.); susic.gordana@gmail.com (G.S.); petrovichristina86@gmail.com (H.P.); 2 Faculty of Medicine, University of Belgrade, 11000 Belgrade, Serbia; bojanbukva@yahoo.com (B.B.); anazek1205@gmail.com (A.C.); majabijelic11@gmail.com (M.B.); tijanatomic1985@gmail.com (T.D.-T.); vlada1309@gmail.com (V.M.); gorandjuricic@gmail.com (G.D.); 3 Department of Pediatric Orthopedic Surgery, University Children’s Hospital, 11000 Belgrade, Serbia; 4 Department of Endocrinology, University Children’s Hospital, 11000 Belgrade, Serbia; smiljka89kovacevic@gmail.com; 5 Department of Pediatric Rheumatology, Clinic of Pediatrics, University Clinical Center, 18000 Nis, Serbia; lazarevic.gaga@gmail.com; 6 Faculty of Medicine, University of Nis, 18000 Nis, Serbia; 7 Institute of Anatomy, Faculty of Medicine, University of Belgrade, 11000 Belgrade, Serbia; 8 Clinic for Rehabilitation “Dr. Miroslav Zotovic”, 11000 Belgrade, Serbia; 9 University Clinical Center of Serbia, 11000 Belgrade, Serbia; adimkic@yahoo.com; 10 Department of Radiology, University Children’s Hospital, 11000 Belgrade, Serbia

**Keywords:** diagnostic imaging, ultrasonography, femur, knee joint, reproducibility of results, adolescents

## Abstract

**Background/Objectives:** CT and MRI are standard modalities for assessing femoral trochlear morphology. Ultrasound may also be useful, as it allows visualization of both the femoral trochlear cartilage and the subchondral bone surface. We aimed to investigate the reliability of ultrasound measurements of femoral trochlear morphology. **Methods:** This was a pilot study including healthy adolescents. Two examiners sequentially performed ultrasound examinations to assess inter-rater reliability, and one week later the first examiner repeated the examination to assess intra-rater reliability. The following measurements were obtained using the contours of the trochlear cartilage and subchondral bone as reference points: sulcus angle, trochlear depth, trochlear facet asymmetry, and ventral trochlear prominence. **Results:** There were 35 participants (16 males and 19 females) with a mean age of 15.1 years (range, 11.1–18.2 years). Inter-rater reliability was moderate to excellent, with ICCs of 0.78–0.89 for bony and 0.67–0.91 for cartilaginous measurements. Intra-rater reliability was also moderate to excellent, with ICCs of 0.66–0.91 for bony and 0.73–0.94 for cartilaginous measurements. Sulcus angle and trochlear depth were the most reliable measurements, followed by ventral trochlear prominence, whereas trochlear facet asymmetry was less consistent. Bland–Altman analysis showed small mean differences and narrow 95% limits of agreement for both inter-rater and intra-rater reliability. **Conclusions:** Sulcus angle, trochlear depth, trochlear facet asymmetry, and ventral trochlear prominence can be reliably measured using ultrasound in healthy adolescents. Overall measurement reliability varies depending on the raters, the specific parameter measured, and whether bony or cartilaginous landmarks are used, but is generally good.

## 1. Introduction

**Figure 1:**
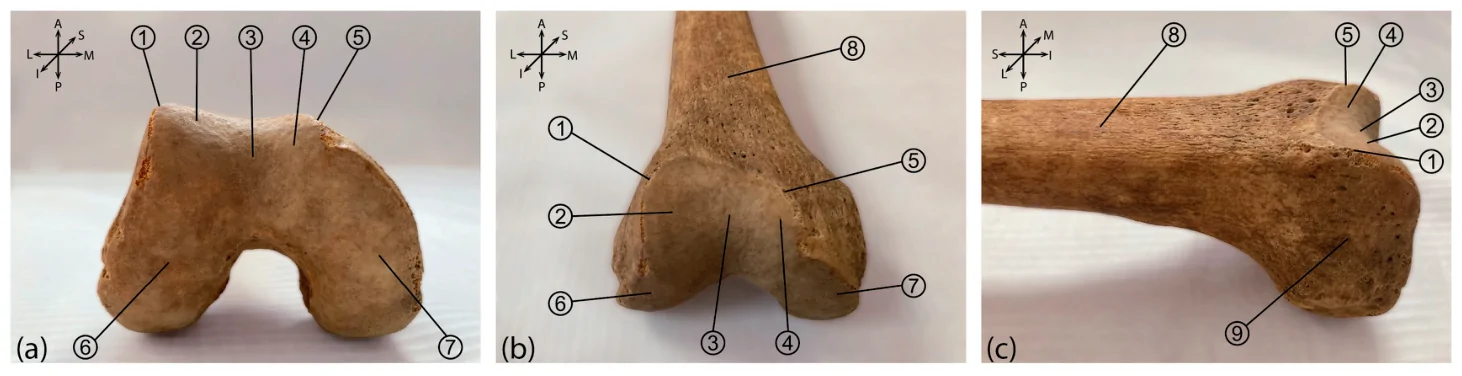
Morphology of the femoral trochlea (patellar surface). Inferior (**a**), anterior (**b**), and lateral (**c**) views of the femoral trochlea: ①—Lateral Trochlear Ridge, ②—Lateral trochlear facet, ③—trochlear sulcus (also called the trochlear groove, intercondylar groove, or floor of the trochlea), ④—Medial Trochlear Facet, ⑤—Medial Trochlear Ridge, ⑥—Lateral femoral condyle, ⑦—Medial Femoral Condyle, ⑧—Distal femoral diaphysis (femoral shaft), ⑨—lateral epicondyle of the femur. Note in (**b**) that the sulcus axis deviates slightly laterally from the anatomical axis of the femur.

The femoral trochlea (patellar surface) is a smooth surface on the anterior aspect of the distal femur (Figure 1), covered with hyaline cartilage. It articulates with the posterior surface of the patella to form the patellofemoral joint. The femoral trochlea consists of two sides (the medial and lateral facets) separated by the trochlear sulcus (also called the trochlear groove, intercondylar groove, or floor of the trochlea), and is bordered by the medial and lateral ridges. The medial and lateral facets form an obtuse angle open anteriorly, called the sulcus angle.

Since the trochlear sulcus is shallower proximally than distally, the sulcus angle is larger proximally than distally [1]. The femoral trochlea is asymmetric, as its lateral facet protrudes more anteriorly, is steeper, and larger than the medial facet [2]. During knee flexion, the patella slides over the trochlea, which stabilizes it and prevents lateral patellar dislocation [3].

If the femoral trochlea is not properly formed, this condition is referred to as trochlear dysplasia. Patients with trochlear dysplasia may experience patellofemoral instability, including subluxation and dislocation of the patella, because the patella is not adequately stabilized during knee flexion [3,4]. The morphological alteration of the most proximal part of the trochlea, which is the shallowest and over which the patella passes at the beginning of the flexion movement, is particularly important [5]. The Dejour classification, used to describe and grade trochlear dysplasia, relies on the assessment of the bony trochlea [6]. However, the evaluation of trochlear cartilage may also be important for several reasons. The patella glides along the trochlear cartilage during knee motion, making its assessment relevant to joint mechanics [4,7,8]. Additionally, in young children, cartilage may provide a more reliable representation of the trochlear shape than bone [9,10]. Furthermore, trochlear dysplasia is believed to result from abnormal early development of the trochlea, which is initially cartilaginous and gradually replaced by bone through the process of endochondral ossification [9,11,12,13].

Radiography and cross-sectional imaging (CT and MRI) have traditionally been used for the assessment of trochlear morphology and the diagnosis of trochlear dysplasia [14]. These imaging modalities, especially CT and MRI, allow the calculation of various quantitative measures of trochlear morphology [5,15,16,17]. Apart from the level at which the trochlea is imaged, trochlear measurements also depend on whether bony or cartilaginous contours are used as landmarks; for example, cartilaginous sulcus angles are typically larger than their bony counterparts [18,19,20].

Ultrasound relies on an adequate acoustic window and has a limited field of view. However, in knee flexion, it allows visualization of both the femoral trochlear cartilage and the surface of the subchondral bone [10,21,22,23]. Therefore, although CT and MRI are considered reference standards for detailed analysis of trochlear morphology, ultrasound may serve as a noninvasive, low-cost, and more readily available alternative. Additionally, ultrasonography is useful in infants and young children because it does not require sedation [12,13,24,25,26]. Since patellofemoral disorders frequently manifest in adolescents, assessing trochlear morphology by ultrasound might be particularly useful in this population [23,27]. Ultrasonography has been shown to be a valid method for assessing the sulcus angle and trochlear depth, as well as the morphology and thickness of the trochlear cartilage [28,29,30]. Some authors, however, have suggested that ultrasonography may not be sufficiently reliable, meaning that ultrasound measurements may show too much variability [13,31].

The aim of this study was to investigate the reliability of ultrasound measurements in the assessment of morphology of the femoral trochlea in healthy adolescents.

## 2. Materials and Methods

For this pilot study, a convenience sample of 35 healthy adolescents seen at a tertiary outpatient rheumatology clinic was selected.

Inclusion criteria were absence of knee complaints, no history of knee injury or surgery, a normal knee physical examination, and signed informed consent.

Patients with rheumatologic or orthopedic knee disorders were excluded from the study. For all participants, the following data were collected: age, sex, body weight, height, and body mass index (BMI).

The minimum sample size was estimated based on the following assumptions: two observations per subject, an intraclass correlation coefficient (ICC) of 0.50 under the null hypothesis and 0.80 under the alternative hypothesis, with α = 0.05 and β = 0.20 [32].

The study was approved by the Ethics Committee of the University Children’s Hospital in Belgrade, Serbia (approval number: 16/237, date of approval: 8 October 2024). Informed consent was obtained from the parents or legal guardians of all participants, and assent was obtained from all participants.

### 2.1. Ultrasound Measurement Protocol

All examinations were performed under the same conditions, on the right knee, using a linear ultrasound probe with a frequency range of 4–12 MHz, on the Acuson NX3 Elite ultrasound system (Siemens Healthineers, Erlangen, Germany). The right knee was selected to ensure a standardized protocol, and measurements were not performed on both knees because they are not independent. Participants were positioned supine, with the leg flexed at a 90-degree angle and the foot resting on the examination table.

**Figure 2:**
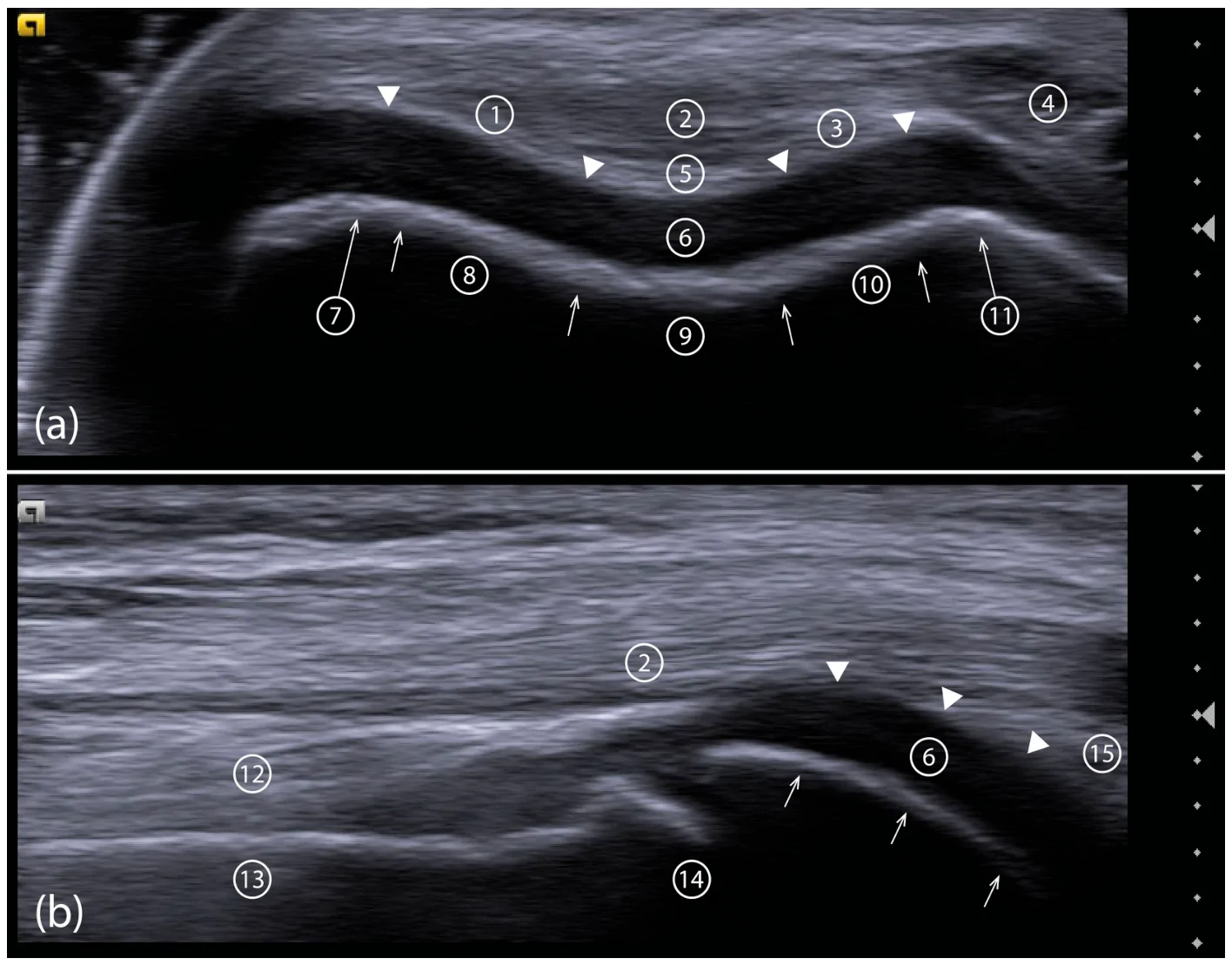
Transverse section of the femoral trochlea (**a**) and longitudinal section of the femoral trochlea through the trochlear sulcus (**b**): cartilaginous contour of the femoral trochlea (arroheads), bony contour of the femoral trochlea (arrows), ①—cartilaginous lateral facet, ②—quadriceps tendon, ③—cartilaginous medial facet, ④—vastus medialis, ⑤—cartilaginous trochlear sulcus, ⑥—trochlear cartilage, ⑦—lateral trochlear ridge, ⑧—bony lateral facet, ⑨—bony trochlear sulcus, ⑩—bony medial facet, ⑪—medial trochlear ridge, ⑫—prefemoral fat pad, ⑬—anterior surface of the distal femoral diaphysis, ⑭—distal femoral epiphysis, ⑮—suprapatellar fat pad.

The femoral trochlea was visualized in two planes: transverse and longitudinal. The probe was initially placed transversely in the suprapatellar region, just distal to the distal femoral epiphysis, perpendicular to the anatomical axis of the femur, and then slightly rotated laterally as needed to align with the orientation of the trochlea (Figure 1b) [8,10]. An image of the most proximal part of the trochlea, which was entirely covered by cartilage, was obtained (Figure 2a).

**Figure 3:**
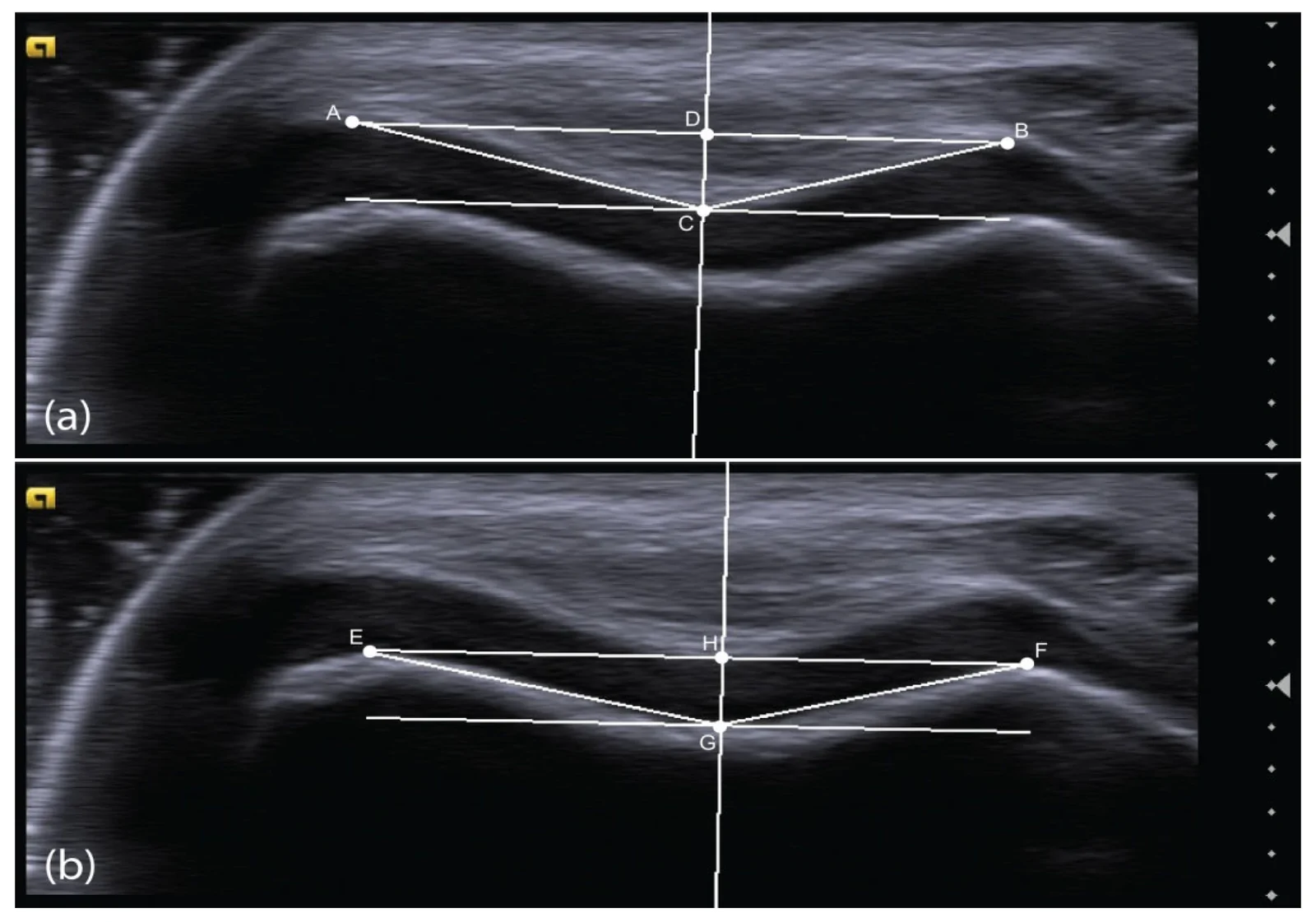
Transverse ultrasonographic images of the femoral trochlea with measurements taken from the trochlear cartilage (**a**) and subchondral bone (**b**). (**a**) Points A and B represent the most anterior points of the cartilage layer over the lateral and medial ridges of the femoral trochlea, respectively. Point C is the lowest point of the cartilaginous sulcus, where a line parallel to line AB touches the concave-up curve of the sulcus. Angle ACB is the cartilaginous sulcus angle. Line segment AC represents the lateral cartilaginous facet, and BC represents the medial cartilaginous facet. The line segment DC, drawn perpendicular to line AB, represents the cartilaginous trochlear depth. (**b**) Points E and F represent the most anterior points of the lateral and medial ridges of the femoral trochlea, respectively. Point G is the lowest point of the bony sulcus, where a line parallel to line EF touches the concave-up curve of the sulcus. Angle EGF is the bony sulcus angle. Line segment EG represents the lateral bony facet, and GF represents the bony medial facet. The line segment HG, drawn perpendicular to line EF, represents the bony trochlear depth.

**Figure 4:**
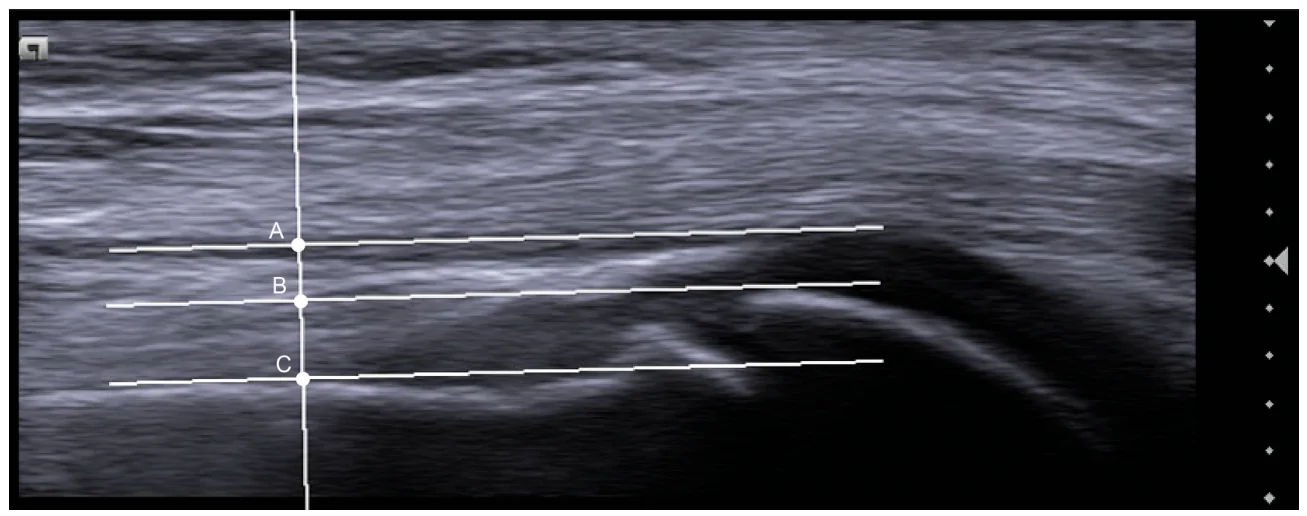
Longitudinal ultrasonographic image through the trochlear sulcus. Ventral trochlear prominence was defined as the perpendicular distance between a line drawn parallel to the anterior surface of the distal femoral diaphysis and a second, parallel line passing through the most anterior point of the trochlear sulcus, either bony (distance BC) or cartilaginous (distance AC).

The probe was then rotated 90 degrees from the transverse position to obtain a longitudinal view through the trochlear sulcus (Figure 2b). From the transverse images, the following measurements were obtained: the sulcus angle, defined as the angle between two lines connecting the lowest point of the trochlear sulcus to the most anterior points of the medial and lateral ridges; trochlear facet asymmetry, calculated as the ratio between the width of the medial and lateral trochlear facets; and trochlear depth, defined as the perpendicular distance from the line connecting the most anterior points of the medial and lateral ridges to the lowest point of the trochlear sulcus. From the longitudinal images, ventral trochlear prominence was measured as the perpendicular distance between a line drawn parallel to the contour of the anterior surface of the distal femoral diaphysis and a second, parallel line passing through the most anterior point of the trochlear sulcus. All the aforementioned measurements were performed using both the contours of the trochlear cartilage and the subchondral bone as reference points (Figure 3 and Figure 4).

The examinations were performed by two ultrasonographers trained in musculoskeletal ultrasound, with at least five years of experience, who had thoroughly reviewed the entire measurement protocol. After the first ultrasonographer completed the examination, the second ultrasonographer repeated the same procedure to assess inter-rater reliability. One week later, the first ultrasonographer repeated the examination using the same protocol to assess intra-rater reliability. To reduce bias, the ultrasonographers were blinded to each other’s measurements. Ultrasound images were exported as TIFF files, and measurements were performed using the ImageJ version 1.54p (National Institutes of Health, Bethesda, MD, USA). Appropriate calibration was applied to ensure consistency with ultrasound measurements.

### 2.2. Statistical Analysis

Numerical data were presented as mean ± standard deviation for normally distributed variables and as median (range) for non-normally distributed variables, while categorical data were presented as frequencies and percentages.

The intraclass correlation coefficient (ICC) was used to assess the reliability of measurements between the two ultrasonographers (inter-rater reliability) and within the first ultrasonographer (intra-rater reliability). ICC values and their corresponding 95% confidence intervals were calculated using a two-way mixed-effects model with a single measurement type (ICC(3,1)) [33]. In both cases, the definition of “absolute agreement” was used. The following criteria were applied for interpreting ICC values: less than 0.50 indicates poor reliability; 0.50–0.75 indicates moderate reliability; 0.75–0.90 indicates good reliability; and greater than 0.90 indicates excellent reliability [33]. ICC was calculated even when the assumption of normality of residuals was not fully met, given the relative robustness of the method to mild deviations from normality.

The Standard Error of Measurement (SEM) was used to estimate measurement error and was calculated using the following formula, $SEM=SD\times \sqrt{1-ICC}$ , where SD is the pooled standard deviation of the measurements, and ICC is the corresponding intraclass correlation coefficient.

Furthermore, agreement between measurements was assessed using Bland–Altman analysis. For inter-rater agreement, the differences between measurements obtained by the two ultrasonographers were plotted against their means. The mean difference (bias), the standard deviation (SD) of the bias, and the 95% limits of agreement (LoAs) were reported. The LoAs were calculated as follows: the bias ± 1.96 × SD of the bias. For intra-rater agreement, the same procedure was applied to repeated measurements performed by the first ultrasonographer. Visual inspection of Bland–Altman plots was used to evaluate systematic bias and outliers. Proportional bias was assessed by performing a linear regression of the differences against the means for each comparison. Data were analyzed using the IBM^®^ SPSS^®^ Statistics software platform (version 17.0; IBM Corp., Armonk, NY, USA).

## 3. Results

### 3.1. Study Participants

In total, 35 participants (16 males and 19 females) with a mean age of 15.1 years (range 11.1–18.2 years) were included in the study. Their demographic data and anthropometric characteristics are presented in Table 1 for descriptive purposes. The mean body weight was 65 kg (range 33.5–102.5 kg), mean body height 168.8 cm (range 150.0–198.5 cm), and mean BMI 22.6 kg/m^2^ (range 14.9–30.4 kg/m^2^).

**Table 1:** Demographic data and anthropometric characteristics of study participants (*n* = 35).

Characteristic	Value
Age (years)	15.1 ± 1.63
Sex, *n* (%)	Male: 16 (45.7%) Female: 19 (54.3%)
Body weight (kg)	65 ± 16.1
Body height (cm)	168.8 ± 10.5
BMI (kg/m^2^)	22.6 ± 3.9

### 3.2. Inter-Rater Reliability

When comparing the first measurements obtained by the first rater with those obtained by the second rater, the inter-rater reliability was moderate to excellent, with ICC values between 0.78 and 0.89 for bony measurements and 0.67 and 0.91 for cartilaginous measurements (Table 2).

**Figure 5:**
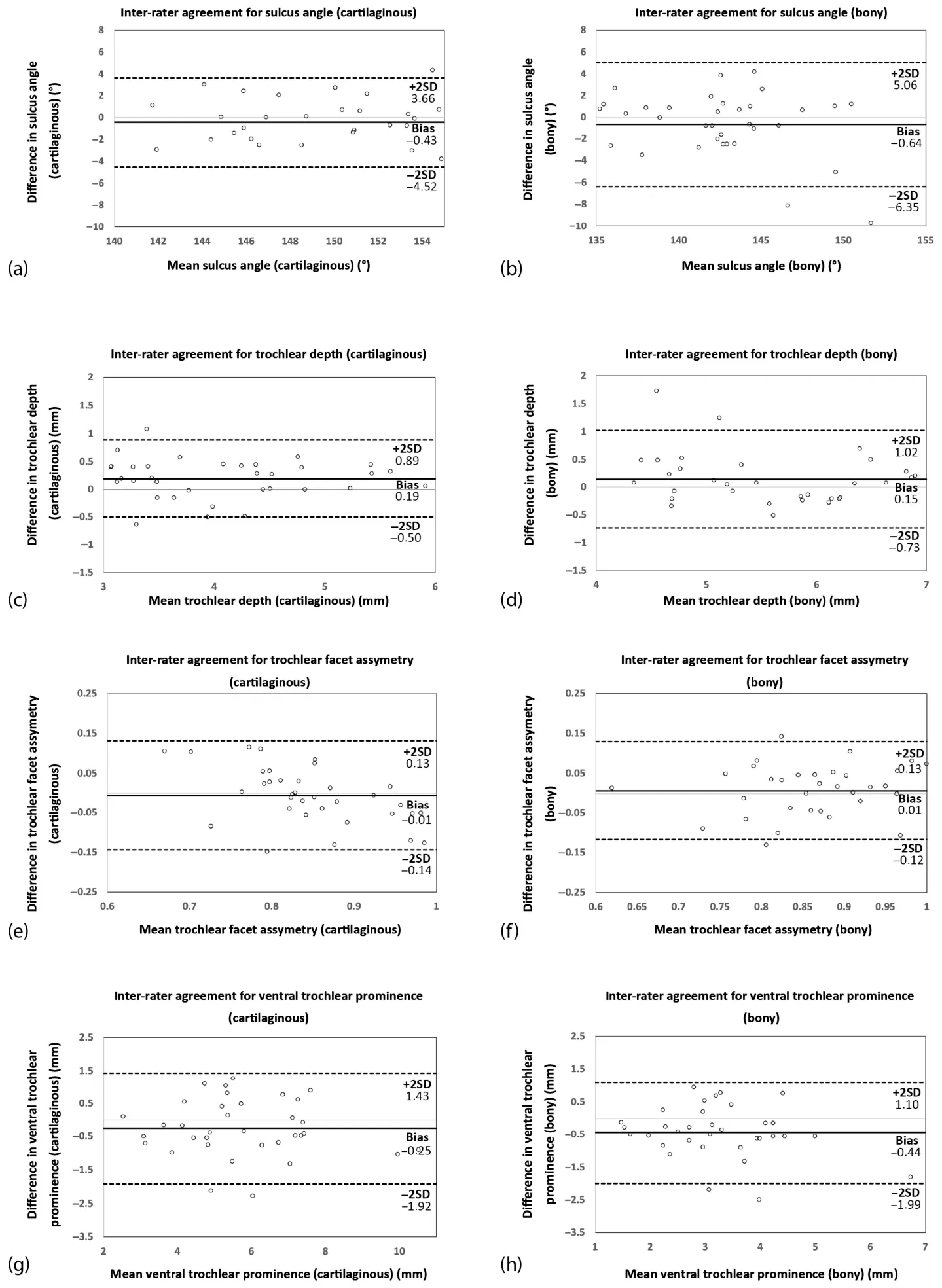
Bland–Altman plots showing inter-rater agreement for eight trochlear measurements: sulcus angle (cartilaginous) (**a**), sulcus angle (bony) (**b**), trochlear depth (cartilaginous) (**c**), trochlear depth (bony) (**d**), trochlear facet asymmetry (cartilaginous) (**e**), trochlear facet asymmetry (bony) (**f**), ventral trochlear prominence (cartilaginous) (**g**), and ventral trochlear prominence (bony) (**h**). The solid line represents the mean difference (bias) and dashed lines represent the 95% limits of agreement.

Bland–Altman analysis demonstrated small mean differences and relatively narrow 95% limits of agreement, suggesting good consistency between raters (Table 3 and Figure 5). The slope for trochlear facet asymmetry measured from the cartilage was significantly different from zero (β = −0.476, *p* = 0.001), indicating proportional bias, with differences decreasing as measurement magnitude increased. No other parameters demonstrated evidence of proportional bias.

**Table 2:** Intraclass correlation coefficients (ICC) with 95% confidence intervals (95% CI), standard error of measurement (SEM), and reliability interpretation for eight femoral trochlear parameters assessed by two raters.

Variable/Measure	Rater 1 Measurement (Mean ± SD or Median (Range))	Rater 2 Measurement (Mean ± SD or Median (Range))	ICC (95% CI)	SEM	Interpretation/Reliability
Sulcus angle (cartilaginous) (°)	150.39 ± 4.75	150.82 ± 5.01	0.91 (0.83–0.95)	0.45	Excellent
Sulcus angle (bony) (°)	141.90 ± 4.80	142.54 ± 5.43	0.84 (0.70–0.91)	0.83	Good
Trochlear depth (cartilaginous) (mm)	4.13 ± 0.85	3.94 ± 0.85	0.89 (0.75–0.95)	0.08	Good
Trochlear depth (bony) (mm)	5.79 ± 0.94	5.64 ± 1.03	0.89 (0.79–0.94)	0.11	Good
Trochlear facet asymmetry (cartilaginous)	0.84 ± 0.07	0.85 ± 0.10	0.67 (0.44–0.82)	0.03	Moderate
Trochlear facet asymmetry (bony)	0.87 ± 0.10	0.87 ± 0.09	0.78 (0.61–0.88)	0.02	Good
Ventral trochlear prominence (cartilaginous) (mm)	5.63 ± 1.79	5.90 ± 1.86	0.89 (0.78–0.94)	0.20	Good
Ventral trochlear prominence (bony) (mm)	3.06 (1.39–6.94)	3.26 (1.53–8.25)	0.79 (0.55–0.90)	0.26	Good

**Table 3:** Bland–Altman analysis for eight femoral trochlear parameters assessed by two raters.

Variable/Measure	Bias ± SD	95% Limits of Agreement		Proportional Bias Slope (β)	*p*-Value for Proportional Bias
		Lower	Upper		
Sulcus angle (cartilaginous) (°)	−0.43 ± 2.09	−4.52	3.66	−0.057	0.457
Sulcus angle (bony) (°)	−0.64 ± 2.91	−6.35	5.06	−0.133	0.196
Trochlear depth (cartilaginous) (mm)	0.19 ± 0.35	−0.50	0.89	−0.005	0.944
Trochlear depth (bony) (mm)	0.15 ± 0.45	−0.73	1.02	−0.097	0.229
Trochlear facet asymmetry (cartilaginous)	−0.01 ± 0.07	−0.14	0.13	−0.476	0.001
Trochlear facet asymmetry (bony)	0.01 ± 0.06	−0.12	0.13	−0.046	0.712
Ventral trochlear prominence (cartilaginous) (mm)	−0.25 ± 0.85	−1.92	1.43	−0.042	0.620
Ventral trochlear prominence (bony) (mm)	−0.44 ± 0.79	−1.99	1.10	−0.183	0.080

### 3.3. Intra-Rater Reliability

The intra-rater reliability was moderate to excellent, with ICC values ranging from 0.66 to 0.91 for bony measurements and 0.73 to 0.94 for cartilaginous measurements (Table 4).

**Figure 6:**
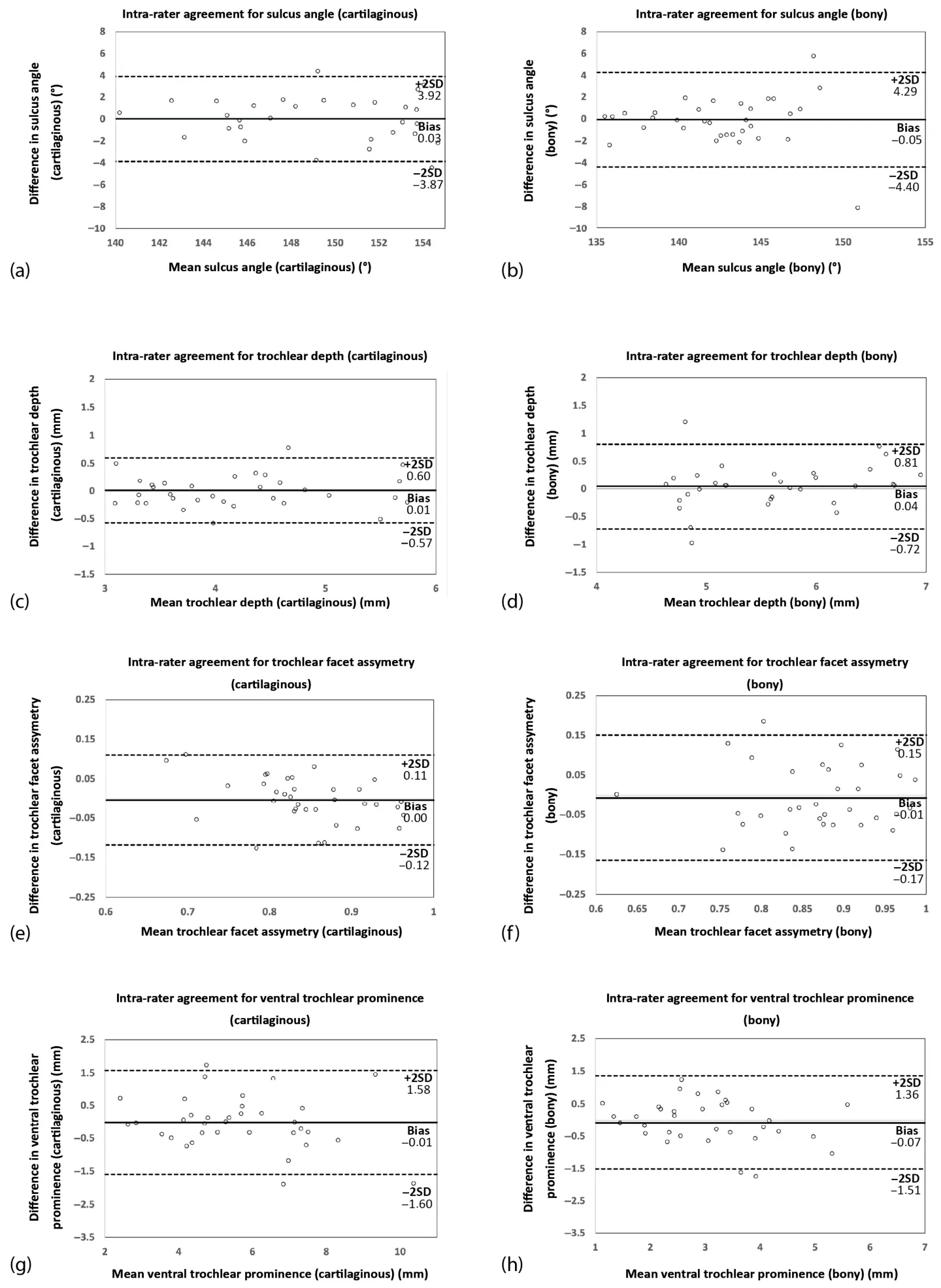
Bland–Altman plots showing intra-rater agreement for eight trochlear measurements: sulcus angle (cartilaginous) (**a**), sulcus angle (bony) (**b**), trochlear depth (cartilaginous) (**c**), trochlear depth (bony) (**d**), trochlear facet asymmetry (cartilaginous) (**e**), trochlear facet asymmetry (bony) (**f**), ventral trochlear prominence (cartilaginous) (**g**), and ventral trochlear prominence (bony) (**h**). The solid line represents the mean difference (bias) and dashed lines represent the 95% limits of agreement.

Bland–Altman analysis revealed small mean differences between repeated measurements by the first rater, with relatively narrow 95% limits of agreement, suggesting good intra-rater consistency (Table 5 and Figure 6). The slope for trochlear facet asymmetry measured from the cartilage and ventral trochlear prominence measured from the bone were significantly different from zero (β = −0.311, *p* = 0.021 and β = −0.242, *p* = 0.007, respectively), indicating proportional bias, with differences decreasing as measurement magnitude increased. No other proportional bias was observed.

**Table 4:** Intraclass correlation coefficients (ICC) with 95% confidence intervals (95% CI), standard error of measurement (SEM), and reliability interpretation for eight femoral trochlear parameters assessed by the first rater on two occasions.

Variable/Measure	Rater 1 Measurement (Mean ± SD or Median (Range))	Rater 1 Repeat Measurement (Mean ± SD or Median (Range))	ICC (95% CI)	SEM	Interpretation/Reliability
Sulcus angle (cartilaginous) (°)	150.39 ± 4.75	150.36 ± 4.93	0.92 (0.84–0.96)	0.40	Excellent
Sulcus angle (bony) (°)	141.90 ± 4.80	141.95 ± 4.99	0.90 (0.81–0.95)	0.50	Good
Trochlear depth (cartilaginous) (mm)	4.13 ± 0.85	4.12 ± 0.86	0.94 (0.89–0.97)	0.05	Excellent
Trochlear depth (bony) (mm)	5.79 ± 0.94	5.74 ± 0.89	0.91 (0.83–0.95)	0.08	Excellent
Trochlear facet asymmetry (cartilaginous)	0.84 ± 0.07	0.85 ± 0.09	0.73 (0.52–0.85)	0.02	Moderate
Trochlear facet asymmetry (bony)	0.87 ± 0.10	0.88 ± 0.10	0.66 (0.42–0.81)	0.03	Moderate
Ventral trochlear prominence (cartilaginous) (mm)	5.63 ± 1.79	5.64 ± 1.97	0.91 (0.83–0.95)	0.17	Excellent
Ventral trochlear prominence (bony) (mm)	3.06 (1.39–6.94)	2.79 (0.86–8.74)	0.87 (0.75–0.93)	0.19	Good

**Table 5:** Bland–Altman analysis of eight femoral trochlear parameters measured on two occasions by the first rater.

Variable/Measure	Bias ± SD	95% Limits of Agreement		Proportional Bias Slope (β)	*p*-Value for Proportional Bias
		Lower	Upper		
Sulcus angle (cartilaginous) (°)	0.03 ± 1.99	−3.87	3.92	−0.040	0.590
Sulcus angle (bony) (°)	−0.05 ± 2.22	−4.40	4.29	−0.039	0.634
Trochlear depth (cartilaginous) (mm)	0.01 ± 0.30	−0.57	0.60	−0.012	0.851
Trochlear depth (bony) (mm)	0.04 ± 0.39	−0.72	0.81	0.052	0.496
Trochlear facet asymmetry (cartilaginous)	−0.00 ± 0.06	−0.12	0.11	−0.311	0.021
Trochlear facet asymmetry (bony)	−0.01 ± 0.08	−0.16	0.15	−0.006	0.971
Ventral trochlear prominence (cartilaginous) (mm)	−0.01 ± 0.81	−1.60	1.58	−0.102	0.184
Ventral trochlear prominence (bony) (mm)	−0.07 ± 0.73	−1.51	1.36	−0.242	0.007

## 4. Discussion

This study evaluated the reliability of ultrasonographic measurements of four cartilaginous and four bony femoral trochlear parameters in healthy adolescents (sulcus angle, trochlear depth, trochlear facet asymmetry, and ventral trochlear prominence). These parameters are well established for assessing trochlear morphology on MRI or CT, but not on ultrasound, although they can also be obtained using ultrasound.

Overall, inter- and intra-rater reliability was high across all parameters. Furthermore, Bland–Altman analysis demonstrated good agreement between measurements. The most reliable parameters appear to be the sulcus angle and trochlear depth, followed by ventral trochlear prominence, whereas trochlear facet asymmetry appears to be the least reliable. The variability in measurements may be explained primarily by the difficulty in obtaining consistent ultrasound sections and identifying accurate reference points on anatomical landmarks. Good ultrasound resolution is also important for clearly delineating the cartilage and bony margins. Furthermore, proportional bias was observed for cartilaginous trochlear facet asymmetry in the assessment of inter-rater reliability, and for cartilaginous trochlear facet asymmetry and bony ventral trochlear prominence in the assessment of intra-rater reliability. However, these findings should be interpreted with caution, as the small sample size may limit the precision of the estimates.

A few studies have assessed the reliability of femoral trochlear measurements obtained by ultrasound [13,25,29,31,34]. However, they differ in study population, sample size, parameters measured, whether trochlear cartilage or the underlying subchondral bone was used as reference points, whether measurements were taken from one or both knees, and so on.

Toms et al. examined the reliability of CT, MRI, and ultrasound measurements of the sulcus angle from the subchondral bone and cartilage in 24 patients with patellar instability (mean age 22 years, range 12–44 years) [31]. Ultrasound measurements of the bony sulcus angle demonstrated reliability comparable to, or slightly lower than, that of MRI and CT, as indicated by the generalizability coefficients. In contrast, sulcus angle measurements taken from the trochlear cartilage were far less reliable than those obtained from CT and MRI. It was found that most of the variance came from the operators acquiring the ultrasound images rather than from the observers measuring the sulcus angles [31]. As the authors have pointed out, achieving good and consistent visualization of the femoral trochlea in patients with patellofemoral disorders may be more challenging and operator-dependent than in healthy individuals, as in our study. However, their results are also not comparable to ours due to several additional sources of variability, including differences in the number of images acquired by the two operators, the use of two observers to measure sulcus angles, and the time interval between initial and repeat measurements. Additionally, the small sample size may have limited the statistical power and precision of the reliability estimates, particularly since only 14 patients participated in the repeat ultrasound examination [31]. Moreover, given that the trochlear sulcus may not be aligned with the anatomical axis of the femur [8], slight lateral rotation of the ultrasound probe may be necessary to avoid distortion of the underlying trochlear morphology. Otherwise, measurements of the femoral trochlea may be inaccurate and unreliable.

An ultrasonographic study of the femoral trochlea in newborns showed that the cartilaginous sulcus angle has low variability and good intra- and inter-observer repeatability [25]. In contrast, trochlear depth measurements were highly variable, making them a less useful parameter for assessing trochlear morphology. However, the authors studied newborns, and the method used to calculate trochlear depth differed slightly from ours, so the results of the two studies are not directly comparable.

Masquijo et al. investigated the intra- and inter-rater reliability of bony trochlear measurements (sulcus angle, trochlear depth, and trochlear facet asymmetry) in 298 infants with a median age of 2 months, of whom 4.4% had developmental dysplasia of the hip [13]. They found moderate to good intra- and inter-rater reliability for the sulcus angle and trochlear depth, but poor reliability for trochlear facet asymmetry [13]. One possible explanation for this is that in infants, the distal femoral epiphysis ossification center is irregularly shaped, making it difficult to assess the bony contour, in contrast to the adolescents included in our study.

Martino et al. investigated the correlation between initial and repeated ultrasound measurements of the bony femoral trochlea (sulcus angle and trochlear depth), performed by a single operator, in 11 healthy male volunteers aged 15–33 years (mean age: 24 years) [29]. The authors found a strong correlation between these measurements [29]. However, correlation does not necessarily imply agreement. Therefore, it cannot be stated with certainty that these ultrasound measurements demonstrate good intra-rater reliability.

In a study of 12 healthy volunteers with a mean age of 24 ± 2 years, the intra- and inter-rater reliability of the bony sulcus measurements was moderate to excellent [34]. The measurements were performed by two raters, one of whom was inexperienced [34]. The intra-rater reliability was lower for the inexperienced rater, further indicating that ultrasound image acquisition and interpretation depend on the skill and experience of the examiner.

Pfirrmann et al. introduced the term ‘ventral trochlear prominence’ as an MRI-based counterpart of Dejour’s trochlear bump (supratrochlear spur) observed on lateral radiographs [5,35,36]. To measure ventral trochlear prominence, they used the most anterior cartilaginous surface of the trochlear sulcus rather than the bony surface as the reference point [5]. Subsequently, Ali et al. used the most anterior bony surface of the trochlear sulcus on sagittal MRI to measure ventral trochlear prominence [15]. We have shown that both the cartilaginous and bony ventral trochlear prominence can also be assessed on ultrasound images. However, it appears that measurements of cartilaginous ventral trochlear prominence are more reliable than those of bony ventral trochlear prominence. This may be at least partly attributed to the irregular bony surface near the distal femoral epiphysis and to the fact that small changes in probe angle or alignment can produce slightly different images in the longitudinal plane. Nevertheless, we believe that the longitudinal ultrasound view of the femoral trochlea may provide additional value in the assessment of trochlear morphology. To the best of our knowledge, no previous studies have evaluated this parameter using ultrasound.

This study has several limitations. First, the small sample size may affect the precision of the estimates and the generalizability of our findings. Second, the raters were trained sonographers who were not randomly selected, which may limit the generalizability of the results to other clinical settings. Third, there are age-specific differences in trochlear morphology that may influence the measurements, and we studied only adolescents [9,10,19,30,37,38]. Finally, patients with patellofemoral disorders were not included, and further studies are needed to evaluate the reliability and validity of ultrasound measurements in this population.

## 5. Conclusions

In conclusion, this study demonstrated that several measurements of femoral trochlear morphology, including sulcus angle, trochlear depth, trochlear facet asymmetry, and ventral trochlear prominence, can be obtained using ultrasound. The reliability of these measurements varies both between two independent raters and within a single rater, additionally depending on whether bony or cartilaginous landmarks are used as reference points; however, overall reliability is generally good. Sulcus angle and trochlear depth showed the highest reliability, followed by ventral trochlear prominence, whereas trochlear facet asymmetry was less consistent. Despite this variability, there was generally good agreement across all measurements. These findings support the reliability of these measurements in the ultrasonographic assessment of both cartilaginous and bony femoral trochlear morphology in healthy adolescents.

Further studies including patients with patellofemoral disorders and comparing ultrasound findings with a reference standard (preferably MRI, which allows detailed visualization of both bone and cartilage) are needed to determine clinical utility.

## Data Availability

Data are available from the corresponding author on request.

## Abbreviations

The following abbreviations are used in this manuscript:

ICC	Intraclass Correlation Coefficient
SEM	Standard Error of Measurement
SD	Standard Deviation
