# Correction: Djordjevic et al. Reliability of Ultrasound Measurements in the Assessment of Femoral Trochlear Morphology in Healthy Adolescents. *Diagnostics* 2026, *16*, 1381

**DOI:** 10.3390/diagnostics16152307

**Published:** 2026-07-23

**Authors:** Stefan A. Djordjevic, Milica Vitomirac, Bojan Bukva, Gordana Susic, Smiljka Kovacevic, Dragana Lazarevic, Ana Cirovic, Hristina Petrovic, Maja Bijelic, Tijana Dimkic-Tomic, Andjela Dimkic-Milenkovic, Vladimir Milenkovic, Goran Djuricic

**Affiliations:** 1 Division of Pediatric Rheumatology, University Children’s Hospital, 11000 Belgrade, Serbia; milicavitomirac@gmail.com (M.V.); susic.gordana@gmail.com (G.S.); petrovichristina86@gmail.com (H.P.); 2 Faculty of Medicine, University of Belgrade, 11000 Belgrade, Serbia; bojanbukva@yahoo.com (B.B.); anazek1205@gmail.com (A.C.); majabijelic11@gmail.com (M.B.); tijanatomic1985@gmail.com (T.D.-T.); vlada1309@gmail.com (V.M.); gorandjuricic@gmail.com (G.D.); 3 Department of Pediatric Orthopedic Surgery, University Children’s Hospital, 11000 Belgrade, Serbia; 4 Department of Endocrinology, University Children’s Hospital, 11000 Belgrade, Serbia; smiljka89kovacevic@gmail.com; 5 Department of Pediatric Rheumatology, Clinic of Pediatrics, University Clinical Center, 18000 Nis, Serbia; lazarevic.gaga@gmail.com; 6 Faculty of Medicine, University of Nis, 18000 Nis, Serbia; 7 Institute of Anatomy, Faculty of Medicine, University of Belgrade, 11000 Belgrade, Serbia; 8 Clinic for Rehabilitation “Dr. Miroslav Zotovic”, 11000 Belgrade, Serbia; 9 University Clinical Center of Serbia, 11000 Belgrade, Serbia; adimkic@yahoo.com; 10 Department of Radiology, University Children’s Hospital, 11000 Belgrade, Serbia

In the original publication [1], there was an error in Figure 3b as published. Specifically, points E, G, and F should all lie along the bony contour of the femoral trochlea. Point G has now been repositioned to lie precisely on the bony contour of the femoral trochlea. The corrected Figure 3 appears below.

The authors state that the scientific conclusions are unaffected. This correction was approved by the Academic Editor. The original publication has also been updated.

## Figures and Tables

**Figure 3:**
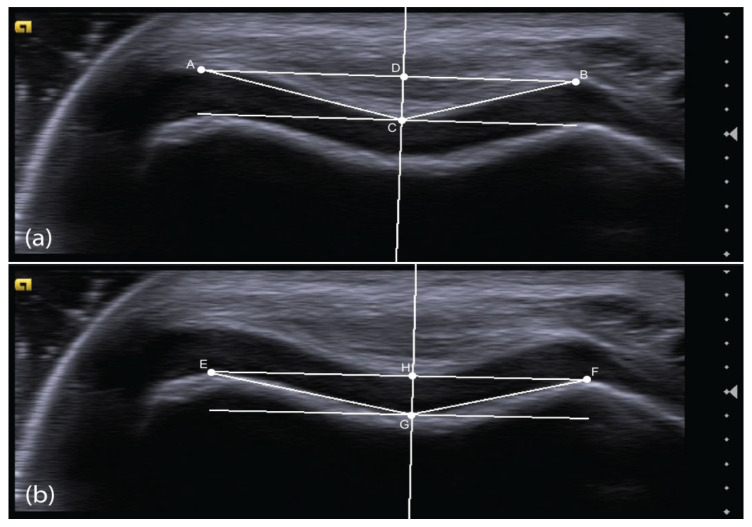
Transverse ultrasonographic images of the femoral trochlea with measurements taken from the trochlear cartilage (**a**) and subchondral bone (**b**). (**a**) Points A and B represent the most anterior points of the cartilage layer over the lateral and medial ridges of the femoral trochlea, respectively. Point C is the lowest point of the cartilaginous sulcus, where a line parallel to line AB touches the concave-up curve of the sulcus. Angle ACB is the cartilaginous sulcus angle. Line segment AC represents the lateral cartilaginous facet, and BC represents the medial cartilaginous facet. The line segment DC, drawn perpendicular to line AB, represents the cartilaginous trochlear depth. (**b**) Points E and F represent the most anterior points of the lateral and medial ridges of the femoral trochlea, respectively. Point G is the lowest point of the bony sulcus, where a line parallel to line EF touches the concave-up curve of the sulcus. Angle EGF is the bony sulcus angle. Line segment EG represents the lateral bony facet, and GF represents the bony medial facet. The line segment HG, drawn perpendicular to line EF, represents the bony trochlear depth.
